# Study on the Filler Composition Optimization and Performance Evaluation of Cold-Patch Asphalt Mixture

**DOI:** 10.3390/ma18163894

**Published:** 2025-08-20

**Authors:** Congwei Bi, Xueqi Wang, Jikai Fu, Hongxu Zhao, Mulian Zheng, Jinghan Xu

**Affiliations:** 1 Shandong Hi-Speed Infrastructure Construction Co., Ltd., Jinan 250098, China; 17660106996@163.com (C.B.); 17753355660@163.com (J.F.); 2 Key Laboratory for Special Area Highway Engineering of Ministry of Education, Chang’an University, Xi’an 710064, China; wangxueqi@chd.edu.cn (X.W.); 13739821837@163.com (H.Z.); 2021021051@chd.edu.cn (J.X.)

**Keywords:** solvent-based cold-patched asphalt mixtures, filler ratio, mixture proportion optimization, cold-patched asphalt slurry

## Abstract

Filler dramatically affects the rheology of cold-patched asphalt (CPA) slurry, as well as the related mechanical properties; its physical and chemical properties will also affect the road performance of cold-patch asphalt mixture (CPAM). In order to optimize the filler composition ratio for CPAM, this study uses an orthogonal test to determine the optimal ratio of bentonite to cement, partially substituting mineral powder. Additionally, a performance verification test suitable for CPAM is designed and performed. The results indicate that the total filler dosage is 4.3%, the proportion of mineral powder replacement is 50%, and the ratio of bentonite to cement is 0.2:1; the forming strength, residual stability, and freeze–thaw splitting strength of CPAM are improved by 7.37%, 20.95%, and 17.13%, respectively, and the water stability is significantly enhanced. Scanning electron microscope images show that the cement and bentonite are dispersed as fillers in CPA, and a hydration reaction occurs, which reveals the mechanism of the optimized cold-patched filler ratio related to performance enhancement.

## 1. Introduction

As road networks become increasingly advanced, the scale of new highways is reduced, and the focus of road construction has been gradually transferred to highway maintenance. Cold-patch asphalt mixture (CPAM) has been widely used in conservation tools because of its all-weather construction, good repair effect, simple construction technology, etc. [1]. It is mainly divided into three categories, including emulsified, solvent, and reactive. Solvent CPAM has become the most widely used variant due to its stable performance, storability, workability, and so on [2]. However, CPAM requires construction at room temperature, which leads to a reduction in the material strength. As the service time of repaired pavement increases, the edges of pothole repairs will become broken and cracked due to traffic load and rainfall, resulting in the failure of the repair material in the pothole and an increase in the severity of secondary damage [3]. At the same time, under high-temperature conditions during the summer, some solvent CPAM repair pavements exhibit poor high-temperature performance, which can lead to rutting and other damage.

Asphalt molecules involved in mineral surface adhesion are much stronger than those involved in intermolecular adhesion; therefore, the asphalt molecules on the mineral surface will be rearranged to form a layer of tight adsorption film, which results in structural asphalt. Outside this film, there is free asphalt between the mineral particles that does not interact with the mineral surface. The surface area of filler is large, reaching 80% of the total surface area of all minerals. After the asphalt slurry forms from the filler and asphalt, the ratio of structural asphalt to free asphalt is significantly affected. Consequently, the nature and amount of filler dramatically impact CPAM performance [4]. Therefore, when enhancing repair results for CPAM, in addition to developing higher-performance solvent-based cold-patched asphalt (CPA) or improving CPAM gradation, optimizing the filler ratio design can also yield better results.

The type and surface area of filler have the greatest impact on the performance of CPAM. The most commonly used filler is limestone mineral powder; however, research shows that adding only mineral powder as filler results in a limited improvement in the performance of CPAM, and selecting the type of filler and optimizing the proportion of blending is a better choice [5]. As early as 1998, Shen [6] summarized that partially replacing mineral powder with finely ground hydrated lime or quicklime powder could effectively enhance the adhesion between asphalt and aggregate, and an optimal dosage of 1–2% was proposed. Li et al. and Li et al. [7,8] used cement to partially replace the mineral powder, and the results demonstrated that this substitution could significantly increase the strength of CPAM. Dong et al. [9] concluded that mixing a certain proportion of cement and bentonite in the filler could effectively improve the water damage resistance of CPAM. Yao et al. [10] used Bayer red mud mixed with mineral powder, which can also improve the water stability, but it exerted a negative effect on the low-temperature performance of CPAM.

Therefore, in order to improve the service life of CPAM and road performance, from the perspective of fillers, studying the filler dosage, filler composition selection, and filler ratios is essential. The three kinds of filler chosen are limestone mineral powder, Portland cement, and bentonite clay. Based on the orthogonal test, the filler ratio will be optimized, which in turn improves the strength, workability, water stability, and temperature performance of CPAM [11,12]. In addition, a scanning electron microscope (SEM) will be used to observe the micro-morphology of the asphalt surface before and after CPAM has been frozen and thawed. Then, the hydration reaction of cement and bentonite dispersed in CPA will be analyzed to reveal the role of an optimized filler ratio in improving CPAM performance at the microscopic level.

## 2. Raw Materials

The fillers can be classified into three categories, including finely ground powders of virgin rocks, test products or by-products, and some waste ashes or finely ground particles of common wastes [13,14,15,16]. Among the three kinds of fillers mentioned above, the first and second types are stored in large quantities and are most widely used, with the best modification effect. Although the third type of filler is in line with the green concept of eco-environmental protection, it is limited by test conditions and higher costs. Therefore, referring to the study by Dong [9], ordinary Portland cement and bentonite composite materials were selected to partly replace the mineral powder in this study.

### 2.1. Asphalt

To ensure that high-performance CPAM exhibits good workability, a base asphalt with low viscosity is typically required. However, in order to guarantee satisfactory performance under high-temperature conditions in the summer, the asphalt must also have sufficient viscosity. So, this study employed SK-90# petroleum asphalt, was produced in Incheon, Republic of Korea, and its key technical indicators are listed in Table 1.

### 2.2. Aggregate and Gradation

Both the coarse and fine aggregates, along with the mineral powder, utilized in this study were limestone and complied with specification requirements, produced in Shaanxi, China. The physical and mechanical indicators of the selected aggregates are presented in Table 2.

**Figure 1:**
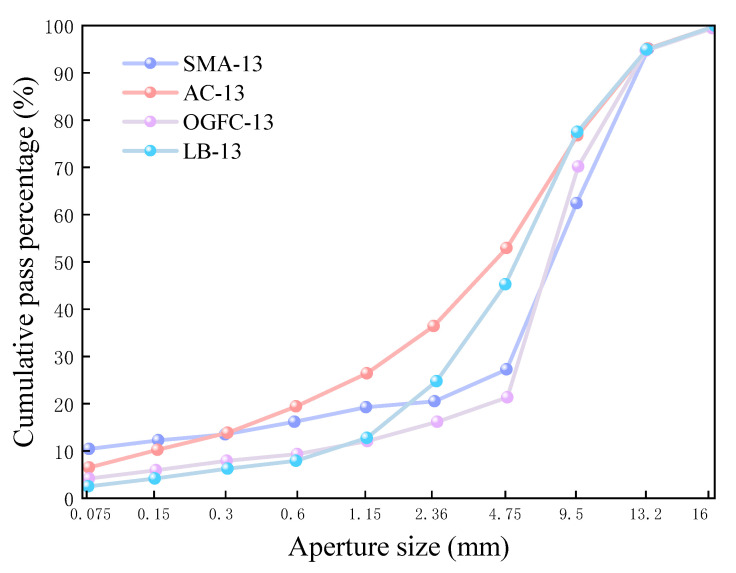
Median curves of different types of gradings.

CPAM is in need of a dense skeleton structure to meet the requirements of high-temperature stability after early traffic and secondary compaction. It is necessary to retain a certain void ratio (greater than 3–5% of the SMA) at the initial stage, so as to facilitate the evaporation of diluent. In the process of utilization, CPAM gradually becomes more dense, the void ratio decreases, the evaporation of diluent also slows down, and stabilized strength emerges. Therefore, the specification (JTG F40-2004) [17] proposed a special LB gradation for both the skeleton compact structure and void ratio of CPAM. The test shows that its early compaction void ratio is about 8–11%. In this paper, with reference to the specification LB-13 gradation and previous research [1], a synthetic gradation is designed, as shown in Figure 1.

### 2.3. Filler

#### 2.3.1. Limestone Mineral Powder

Limestone mineral powder offers advantages such as a large specific surface area, excellent stability, and high alkalinity. As the fundamental filler component, its properties must meet the specification requirements. This study utilized S95 limestone mineral powder from the Shaanxi JingHe Factory, whose relevant mechanical indicators are presented in Table 3.

#### 2.3.2. Portland Cement

When cement is added to CPAM as a filler, it firstly reacts with the aggregates, forming compounds that enhance the bonding strength between the aggregates and asphalt. Secondly, the alkali components such as Ca(OH)_2_ released during cement hydration undergo a pozzolanic reaction with SiO_2_ in mineral slag, generating denser hydration products that improve the asphalt–aggregate adhesion. Concurrently, the exothermic hydration process elevates the temperature, promoting volatilization of the diluent in the CPAM. This accelerates the restoration of asphalt viscosity and properties, facilitating strength development. P.O 42.5 cement from the JiDong Cement Factory was used, with key technical indicators listed in Table 4.

#### 2.3.3. Bentonite

**Figure 2:**
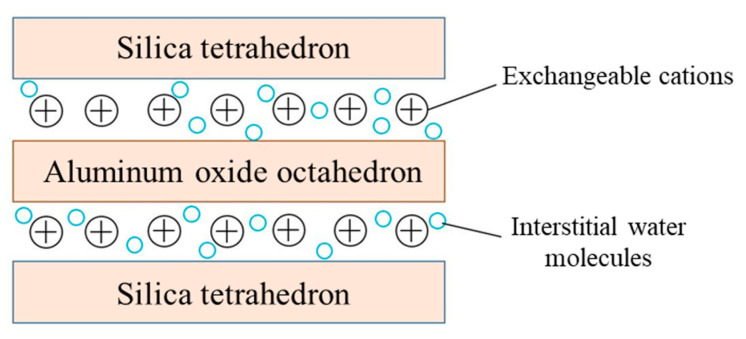
Bentonite layered junction structure.

Bentonite is a mineral containing 85–90% montmorillonite, renowned for its exceptional water absorption capacity. Upon water contact, bentonite could expand 20 to 30 times in volume into a paste state. Montmorillonite ((Na, Ca)_0.33_(Al, Mg)_2_[Si_4_O_10_](OH)_2_·nH_2_O) features a sandwich-like layered structure: two silica tetrahedral sheets enclosing an alumina octahedral sheet. A weak interlayer bonding force allows cations within its crystalline structure to be readily exchanged with others, resulting in high ion-exchange capacity and significant water absorption swelling, and the structural model is shown in Figure 2 [9].

Relevant studies have shown that the effect of sodium-based bentonite is much better than calcium-based bentonite [20]. When bentonite is added to asphalt as a filler, the mixture can absorb water and expand after undergoing rain erosion to close voids, providing protection for CPAM to resist water damage. Therefore, a sodium-based bentonite was selected in this study. The basic physical measurement results of the bentonite are shown in Table 5.

## 3. Test Methods

### 3.1. Orthogonal Test

The total filler amount, proportion of bentonite and cement replacing mineral powder, and the ratio of bentonite and cement are the three most important factors of the filler that affect the performance of CPAM. Therefore, an L_9_(3^3^) orthogonal test is used to design the optimum group by using the three factors as Factors A, B, and C, and selecting three levels for each factor.

Through investigation, it is found that when using cold-patching materials (CPAM) in the United States, the amount of mineral powder is relatively high, up to 10%; on the contrary, in Canada, the amount of mineral powder used is very small or even not used, usually 2% or less; while the recommended upper limit of mineral powder content for CPAM in Chinese specifications is 5% [21]. Usually, the more filler used with the same oil–stone ratio, the worse workability and the better strength. According to the existing research results of our research group, a 4.3% mineral powder dosage of CPAM can obtain better compatibility, but it also meets the other performance requirements. So, the mineral powder dosage uses the 4.3% as the median value, and 2% as a gradient to select the 2.3%, 4.3% and 6.3% as the three dosage levels. Therefore, we have chosen filler content levels with a relatively large span, which not only covers the common content ranges in multiple countries to ensure the completeness of performance investigation under a large span but also ensures that it does not exceed the domestic recommended limit by too much.

Finally, based on the commonly used ratio, 30% of bentonite and cement was selected as the median value with reference to Maha’s study [22], and 10%, 30% and 50% were determined with a gradient of 20%. Referring to Katsioti et al. [23] and Dong et al. [9], 0.2:1, 0.6:1, and 1:1 were selected as the three dosage levels of Factor C.

### 3.2. Range Analysis and Analysis of Variance (ANOVA)

In order to examine the effect of each factor on the CPAM performance, the results of the orthogonal tests were specifically judged using extreme variance analysis to determine the order of influence of each factor on performance. Range analysis can obtain the degree of influence of different factors on the test index, which is also called sensitivity analysis. ANOVA can obtain the size of the experimental error. Ultimately, the results of the extreme variance analysis and ANOVA were used to determine the degree of each factor’s significance, which in turn determined the optimal solution.

### 3.3. Performance Verification Tests for CPAM

For determining the optimal ratio of filler in CPAM, except for variance analysis and range analysis of the orthogonal test results to determine each factor’s significance, this study also used a single factor analysis to illustrate the performance enhancement effect of CPAM more intuitively. The methods of determining the response indexes for the single factor analysis are as follows. The equipments used in the test process should meet the corresponding specifications and requirements, and there is no specific model.

#### 3.3.1. Strength Test

1. Early strength

The test method for the early strength of CPAM is not given in JTG F40-2004 [17]. Therefore, we mainly refer to the hot-mix asphalt mixture. The test steps are as follows:

(1) The mixed CPAM was kept at a room temperature of 20–25 °C for 6 h; then, about 1170 g of CPAM was weighed and loaded into the Marshall mold at room temperature (the specific weight was 63.5 mm ± 1.3 mm after the Marshall specimen was compacted).

(2) The Marshall compaction instrument was used to compact 50 times, double-sided, and then cooled at room temperature for 2 h; then, the specimen was demolded.

(3) The Marshall stability tester was used to perform vertical loading at the rate of 50 mm/min until the specimen was destroyed, measuring the stability, which should be greater than or equal to 2 kN. This method was used to evaluate the early strength of CPAM.

2. Forming strength

**Figure 3:**
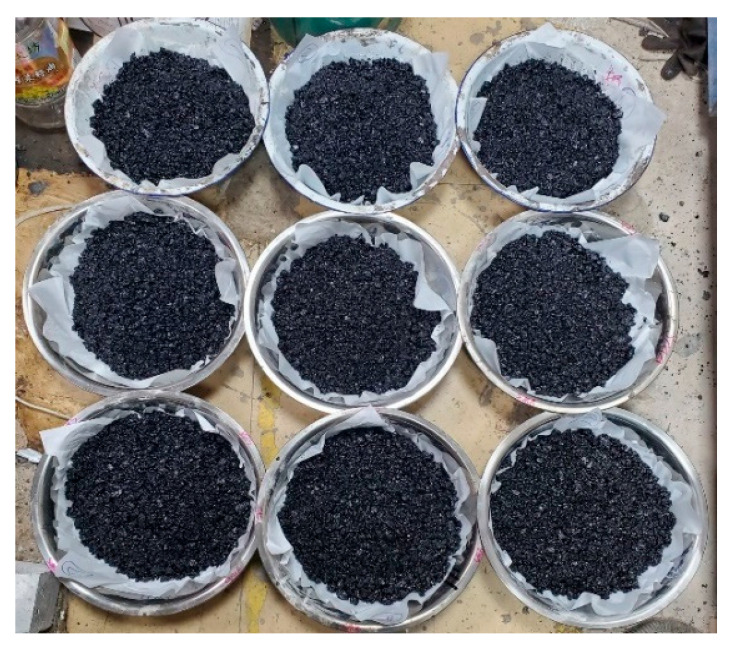
CPAM with different filler ratios.

The forming strength was tested according to the JTG F40-2004 [17] test method. The mixed CPAM was spread into the vessel and put into the 110 °C oven conserving for 24 h; then, it was loaded into the Marshall compaction instrument and compacted 75 times, double-sided, to prepare the Marshall specimen. After, the specimen was cooled, demolded, and placed in a ventilated environment for 1 d. As for the modified water bath temperature in the immersion Marshall test, the specimen was placed in a 40 °C water bath box to keep warm and wet for 30 min; then, we used the above method to measure the stability. The stability should be greater than or equal to 7 kN. The CPAM with different filler ratios is shown in Figure 3.

#### 3.3.2. Construction Workability Test

Although the empirical method of JTG F40-2004 [17] can distinguish the workability of CPAM under low-temperature conditions, the CPAM generally has good workability at room temperature; thus, this empirical method is not suitable for distinguishing different types of CPAM.

**Figure 4:**
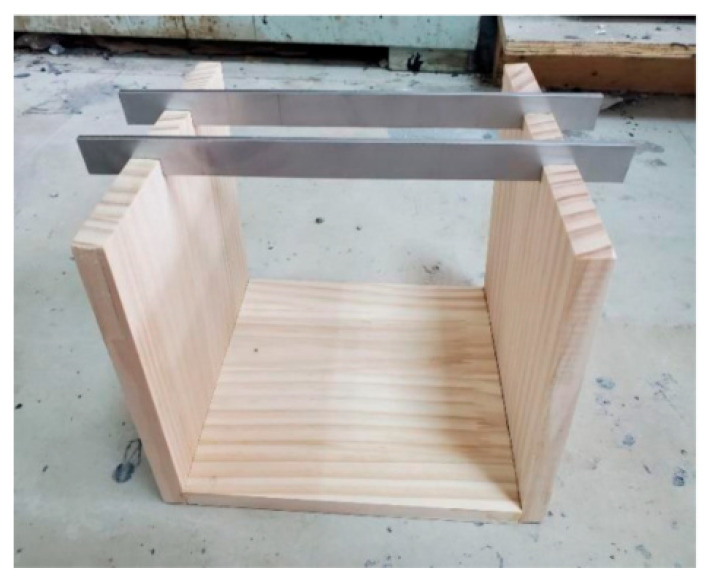
Workability U-shaped frame.

Therefore, this paper followed the standard recommendation method for the low-temperature workability evaluation of CPAM. The workability testing method at room temperature referred to the method proposed by Xu [24], with local correction, called the self-weight dispersion method. The test steps are as follows:

(1) Firstly, 800 g of CPAM was prepared in the mold at room temperature; then, the Marshall compaction instrument was used to compact the specimen 5 times, double-sided, and the specimen was kept at 25 °C for 5 h.

(2) Then, a U-shaped frame composed of three 200 mm × 200 mm wooden boards was prepared, where both sides of the frame had slots for placing the blade boards, with a distance of 50 mm between the blade boards; the instrument is shown in Figure 4.

(3) After demolding, the CPAM was put on the edge plate of the U frame. The time when the specimen was completely scattered was recorded. Each orthogonal group was tested three times, and the average value was taken.

When the scattering time of CPAM is less than 80 s, it exhibits excellent workability and ease of construction.

Then, the grades of the CPAM’s workability at room and low temperatures in accordance with the empirical method of JTG F40-2004 [17] were judged; the indexes should be higher than at least grade 5 at 25 °C and at least grade 3 at −5 °C.

#### 3.3.3. Immersion Marshall Test

Referring to the method of JTG F40-2004 [17], we found that when this method was used in the CPAM test, the 60 °C water bath temperature requirement for the hot-mix asphalt mixture was too harsh, the strength loss of CPAM was serious, and some specimens even appeared loose. Therefore, combined with the previous achievements of the research group [1], the water bath temperature of the experimental group was modified to 40 °C. The Marshall stability of the specimen was measured by the Marshall stability tester after 48 h of insulation under the modified water bath temperature.

In order to ensure a consistent maintenance environment with the control group, the specimens were maintained but not submerged under the condition of 40 °C for 48 h, and then, after 40 °C immersion for 30 min, the Marshall stability was determined. Finally, the water stability of CPAM should be evaluated by calculating the residual stability, which should be at least 85% higher.

#### 3.3.4. Freeze–Thaw Splitting Test

JTG F40-2004 [17] includes the freeze–thaw splitting test method of hot-mix asphalt mixture; however, for the solvent-based CPAM, it could not be completely copied. The water bath temperature was the same as for the immersion Marshall test. Because the frozen group specimens need to be cured in a constant temperature water bath for 24 h after freezing, the unfrozen group specimens were also placed in a 40 °C water bath for 24 h. The test steps were as follows:

(1) The Marshall specimens were formed according to the forming strength, demolded, and divided into two groups.

(2) The first set of specimens was placed in a 40 °C oven for 24 h and set aside for later use. The second set of specimens was vacuum saturated, subject to the standard saturation test and placed in plastic bags. Then, 10 mL of water was added to the bags before being sealed, and the specimens were placed in a refrigerator at −18 °C ± 2 °C for 16 h. After freezing, the specimens were immediately placed in a water tank at 40 °C ± 0.5 °C for 24 h.

(3) According to JTG F40-2004 [17], both groups of specimens were placed in a 25 °C water bath for 2 h and removed to obtain the splitting tests. The Marshall stability tester was used at the rate of 50 mm/min until the specimen was destroyed, and the splitting tensile strength of the specimen was recorded. The effective average splitting strengths of both specimen groups were measured, and the freeze–thaw splitting strength ratio (TSR) was calculated, which should be at least 80% higher.

#### 3.3.5. Rutting Test

It is not appropriate to evaluate the high-temperature stability of CPAM by using the dynamic stability at 60 °C used in the traditional asphalt mixture. The diluent cannot be completely volatilized in the process of the curing of CPAM. When the test temperature is 60 °C, the specimen will become soft, the dynamic stability will be small, and the deformation of the specimen will be large. Therefore, combined with the modified water bath temperature of water stability above, the modified rutting test temperature was 40 °C. The test steps were as follows:

(1) The CPAM was loaded into the rutting plate mold and then preliminarily shaped with a wheel miller 4 times at room temperature.

(2) Then, the CPAM was put into the oven at 110 °C for 24 h and rolled with the wheel miller at 110 °C 8 times to form the rutting test specimen. The specimens were put into a ventilated environment at room temperature to be cooled for at least 12 h before demolding; then, they were maintained for 3 d.

(3) According to the 40 °C rutting test requirements (JTG E20-2011) [25], the dynamic stability was selected as an indicator to evaluate the high-temperature performance of CPAM, which should be at least 3000 higher.

#### 3.3.6. Low-Temperature Bending Test

The low-temperature cracking resistance of CPAM can be evaluated by the hot mix material evaluation method, according to T0715-2011 in JTG E20-2011 [25]. The rutting plate specimen was molded according to the same method presented in Section 3.3.5, and the molded rutting plate was cut into standard-size trabecular specimens, according to the method T0715-2011 in JTG E20-2011 [25]. The specimens were put into the −10 °C environmental chamber for 45 min, and the universal testing machine was used to carry out the low-temperature bending test, calculating the bending and tensile strains, bending and tensile strengths, and bending modulus at the time of specimen destruction. Evaluating the low-temperature performance of CPAM, the bending and tensile strain should be higher than at least 2800 μƐ.

### 3.4. Scanning Electron Microscope (SEM) Imaging

In order to analyze the degree of hydration reaction between cement and bentonite dispersed in CPA and reveal the mechanism of the optimized cold patch filler ratio on the performance enhancement at the microscopic level, SEM, model SU-8010 (Tokyo, Japan), was used to observe the microscopic morphology of the CPA surface before and after freezing and thawing. Due to the size requirements of the specimens, CPAM with a thin thickness and size within 1 cm was taken out from the Marshall specimens, and the magnified images were obtained at 500 times, 2000 times, and 5000 times.

## 4. Results and Discussion

### 4.1. Orthogonal Test Results

The orthogonal test combination table design is shown in Table 6. The actual amount of the three fillers to be used in each group was calculated according to Table 7.

The CPAMs were mixed according to different ratios for each group and tested for strength, workability, water stability, and temperature properties according to the test methods described above, and the test results are summarized in Table 8.

### 4.2. Range Analysis and ANOVA

*K_i_* indicates the sum of the test results of a factor at three levels, and *k_i_ = K_i_*/3, which indicates the average of the single factor test results at three levels; the range, *R* = max{*k*}-min{*k*}, where the larger the R value is, the greater the impact of this factor on the indicator will be. The results of the range analysis are shown in Table 9 and Table 10.

The results show that in terms of strength, factor A tops the list, followed by factor C; in terms of workability, factor A also had the greatest impact, followed by factor B; for the residual stability, factor A was the most prominent, but the TSR was most affected by the factor B; the temperature performance was also dominated by the influence of factor A, followed by factor B, and factor C was the smallest. Comprehensive analysis shows that the total filler amount was the core variable that determined the comprehensive performance of CPAM. Factor B had the greatest effect on the TSR, indicating that part of the mineral powder replaced by cement–bentonite can effectively improve the water stability of the material. Factor C was second only to factor A in strength sensitivity, but its influence was weaker than that of factors A and B in all other performance indicators.

When performing ANOVA on factors and levels, a blank column is needed to perform as an error column. When the mean square of one of the three factors is smaller than the error mean square, it is necessary to recalculate the mean square after combining the mean square and the freedom degrees of that factor to the error and then perform the ANOVA again. The effect significance of a factor could be shown by the degree of freedom (*d_f_*), mean square deviations (*MS*), test statistics (*F*), and probability level (*P*), as shown in Table 11.

The results of ANOVA show that factor A significantly affected the construction workability and temperature performance of CPAM and relatively affected the early strength, while factors B and C had no significant effect on the early strength and forming strength. In terms of the high-temperature performance, factor A had a highly significant effect on the dynamic stability, and factors B and C had no significant effect. In terms of water stability and low-temperature cracking resistance, factors A and B had the greatest impact on the residual stability and damage bending and tensile strains. Factor C, the proportion of cement and bentonite replacing the mineral powder, affected the low-temperature breaking bending and tensile strain. The effects of ANOVA on the significance of each factor are shown in Table 12.

### 4.3. Performance Verification of CPAM

#### 4.3.1. Strength

**Figure 5:**
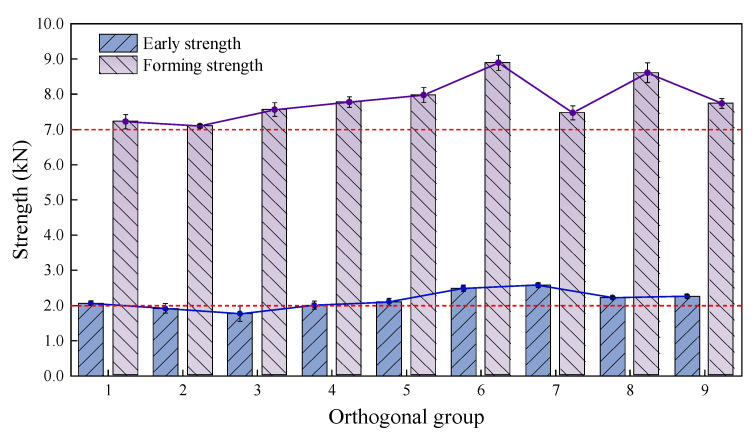
Early strength and forming strength of orthogonal groups.

The intuitive analysis of different orthogonal groups’ strengths is shown in Figure 5.

**Figure 6:**
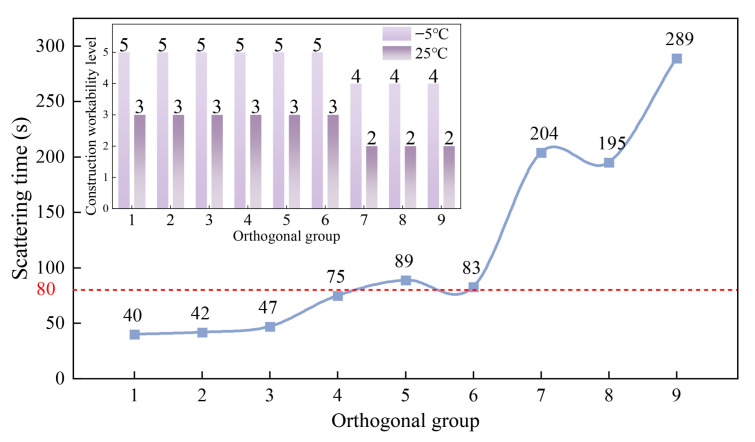
Orthogonal group construction workability.

**Figure 7:**
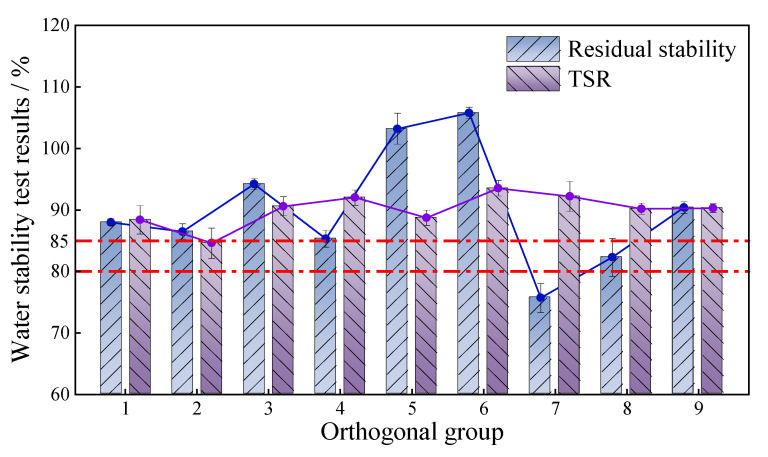
Orthogonal group water stability test results.

**Figure 8:**
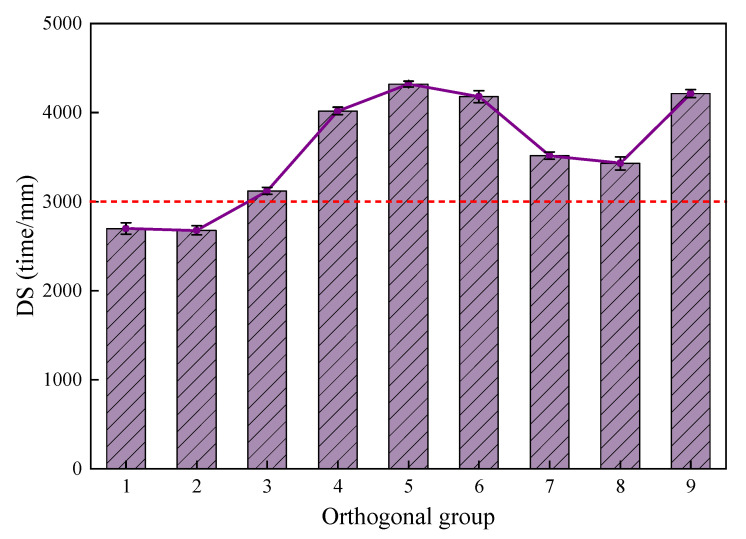
Orthogonal group dynamic stability.

**Figure 9:**
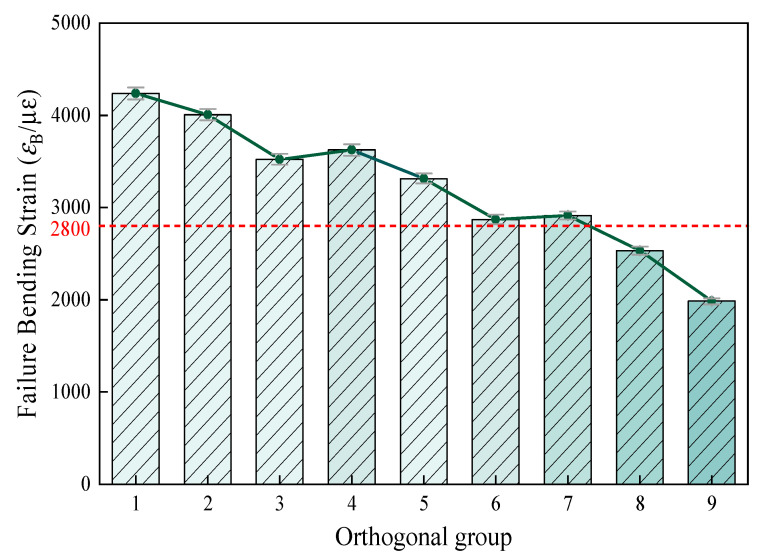
Orthogonal grouping failure bending strains.

The red lines in Figure 5, Figure 6, Figure 7, Figure 8 and Figure 9 represent the index requirement specified in the test methods. The results show that only the early strengths of groups 2 and 3 (1.92 kN and 1.77 kN, respectively) did not meet the requirements. Groups 6 and 7 had relatively high early strengths of 2.49 kN and 2.58 kN, respectively. Groups 5, 6, and 8 had greater forming strengths of 7.98 kN, 8.89 kN, and 8.61 kN, respectively. However, the strengths of groups 1, 2, and 3 were relatively low, but the early strength and forming strength of group 1 met the requirements. This is because the total filler amount has a significant impact on the early strength, and a low filler amount will increase the free asphalt content in the CPAM. Therefore, groups 1, 2, and 3, with a filler amount of only 2.3% had relatively low early strengths. Groups 7, 8, and 9 had the highest filler amounts and relatively higher initial strengths, but the forming strengths of groups 7 and 9 were lower.

This indicates that when the total filler amount is the same, the ratio of bentonite and cement replacing mineral powder at 30% and a bentonite/cement ratio of 0.2:1 could achieve higher strength, which was group 8. Meanwhile, group 9 used a ratio of 50% bentonite and cement replacing mineral powder and a bentonite/cement ratio of 1:1, which did not reach the highest strength. This also proves that it is not necessarily true that a higher content of cement and bentonite is better; excessive cement or bentonite may reduce the cohesion of the asphalt binder, and thus, the strength of the CPAM would decrease. Considering all factors, group 6 had the highest initial and forming strengths, followed by groups 8, 5, and 1 with better strength.

#### 4.3.2. Construction Workability

The workability analysis of different orthogonal groups is shown in Figure 6.

Based on the construction workability and scattering time, the lowest filler amount of groups 1, 2, 3 had the shortest scattering time of only 40 s, 42 s, and 47 s, which were far better than the stipulated scattering time of less than 80 s. Groups 4, 5, and 6 had 4.3% moderate, and the scattering time was approximately 80 s, which barely met the requirements. While when the filler amount reached 6.3%, the scattering time of groups 7, 8, and 9 increased sharply, reaching 204 s, 195 s, and 289 s. The low-temperature construction workability grades of these three groups were only grade 2, which did not meet the basic requirements for low-temperature construction. The construction workability at room temperature grade was only grade 4, which did not meet the requirements of grade 5. This indicates that the more filler used, the longer the scattering time, and the worse the workability. Among all groups, group 9 had the longest scattering time and the worst workability. The reason might be that the proportion of bentonite and cement replacing the mineral powder in group 9 was the largest, and the amount of bentonite was the most. Therefore, it is concluded that when the total filler amount is the same, the larger the proportion of bentonite and cement replacing the mineral powder, the worse the workability will be.

#### 4.3.3. Water Stability

The analysis of the immersion Marshall test and freeze–thaw splitting test results is shown in Figure 7.

From the test results, it was seen that except for group 7 and group 8, all other groups met the requirement of residual stability (≥85%). Group 5 and 6 showed a residual stability greater than 100%, indicating that hydration of cement took place during the immersion of the specimen, thus increasing the strength. Group 7 had the worst residual stabilization at a filler amount of 6.3%. This suggests that excessive filler content and too thin an asphalt film led to a decrease in the internal cohesion of the mixture, making the mixture more susceptible to water erosion. Group 8 and 9 both showed a significant improvement in residual stability compared to group 7, with group 9 achieving a residual stability of 90.4%. This indicates that increasing the proportion of cement and bentonite could significantly improve the water stability of CPAM, and a greater bentonite/cement ratio could gain a more significant performance improvement. Based on the test results, the groups with the best residual stability were groups 5 and 6, followed by groups 3 and 9. The TSR of all orthogonal groups met the requirement of >80%. Similarly, group 6 had the highest TSR, reaching 93.5%, while group 2 had the lowest TSR at 84.6%. The TSR of the other groups also showed significant improvement, and the analysis of variance indicated that the total filler content, the proportion of the two fillers, and the ratio of bentonite to cement all had a significant impact on the TSR. This shows that the addition of cement and bentonite can to some extent improve the water stability of CPAM. However, based on the results of the water immersion Marshall test, the greater addition of cement and bentonite is not necessarily better.

Above all, group 6 had the best water stability, followed by group 3, group 5, and group 9, with good residual stability and TSR.

#### 4.3.4. High-Temperature Stability

The analysis of the high-temperature stability is shown in Figure 8.

The ANOVA indicates that only the total amount of filler had a significant impact on the dynamic stability; factors B and C had no significant effect. From Figure 8, it is intuitively seen that the filler amounts in CPAM corresponding to the groups 1, 2, and 3 were the lowest, and the dynamic stability was relatively low. The reason is that less filler and more free asphalt in the mixture would result in a lubricating effect between the mixtures, which makes the surface of the mixture more prone to rutting, and the dynamic stability decreases. When the filler amount was increased to 4.3% and 6.3%, no significant improvement in dynamic stability was observed, and the peak value was concentrated in groups 4, 5, and 6 with a moderate filler amount. This indicates that it is not the case that the higher the filler amount, the better the high-temperature performance of the CPAM. Group 9, with a higher proportion of bentonite and cement, showed a significant improvement in high-temperature performance, indicating that bentonite and cement can conspicuously improve the high-temperature performance of the CPAM. Considering all factors, groups 5, 9, 6, and 4 had the best high-temperature performance.

#### 4.3.5. Low-Temperature Cracking Resistance

The analysis of the low-temperature cracking resistance is shown in Figure 9.

The results of the ANOVA indicate that the total amount of filler and the proportion of cement and bentonite replacing the mineral powder had extremely significant effects on the low-temperature cracking resistance of CPAM. As can be seen from Figure 9, with the increase in filler dosage, the failure bending strain decreased significantly. When the filler amount is the same, a higher the proportion of cement and bentonite replacement can be, and a lower failure bending strain of CPAM can be achieved. This change trend can clearly be observed from the test results of groups 1 to 9. Most groups met the requirement of failure bending strain. When the filler amount reached 6.3%, the failure bending strain of each group significantly decreased, which in group 9 was only 1984 Ꜫ_B_/μꜪ, with the most significant decrease. Although the low-temperature failure bending strain of group 6 also decreased, it still met the requirement of ≥2800 Ꜫ_B_/μꜪ specified in the standard.

### 4.4. Determination of the Optimal Ratio

Combining the test results of the single-factor ANOVA, groups 1, 2, and 3 exhibited poor early strength and high-temperature performance, even failing to meet the requirements. Groups 7, 8, and 9 had poor construction performance, which did not conform to the construction workability grade requirements. Therefore, groups 4, 5, and 6 were compared to determine the group with the optimal comprehensive performance, and the comparison results are shown in Table 13.

From the comparison results in Table 13, it can be concluded that the CPAM of group 6 has the best strength and water stability. In addition, the construction workability and high-temperature performance of groups 4, 5, and 6 are close. However, the low-temperature performance of group 6 is the worst, but it still meets the application requirements. Comprehensively considering the pavement performance of CPAM in different groups, group 6 performs well in all other aspects except for slightly poor low-temperature performance. Therefore, the CPAM group with the optimal comprehensive performance is group 6: the total amount of filler is 4.3%, the ratio of bentonite to cement is 0.2:1, and 50% of the mineral powder is replaced.

**Figure 10:**
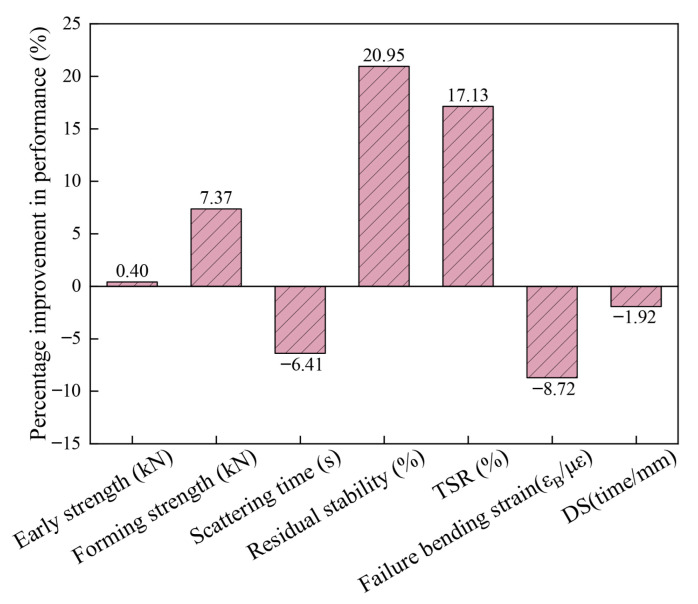
The performance improvement percentage of group 6 compared to the conventional group.

On this basis, the performance of the conventional group and group 6 were compared. The total amount of filler in the conventional group is 4.3%, and the filler is 100% lime powder. Table 14 shows the performance comparison between group 6 and the conventional group. The percentages of improvement in each performance are shown in Figure 10.

By contrast, when using the self-developed CPAM with a total filler amount of 4.3%, replacing 50% of the mineral powder with bentonite and cement, and with a ratio of bentonite to cement at 0.2:1, the strength and water stability of the CPAM can significantly improve, showing an increase of 20.95%, 17.15%, and 7.37% in residual stability, TSR, and molding strength. The improvement mechanism is that the hydration of cement and the pozzolanic reaction enhance the structural strength under water immersion conditions, while the water absorption expansion of bentonite effectively seals the fine pores. However, this combination only increased the early strength by 0.4%, mainly due to the softness of the initial asphalt slurry. The strength is mainly provided by the mineral material interlocking, and the improvement effect of the adhesive force of the slurry is not yet significant. On the other hand, although this modification had certain negative impacts on construction workability, low-temperature cracking resistance, and high-temperature performance, the variation ranges were small and all met the specification requirements; so, the negative impacts were within the controllable range.

Considering that the temperature performance of the CPAM in group 6 was not the best, in addition to recommending it as the group with relatively optimal comprehensive performance, we have supplementary suggestions on CPAM types more suitable for specific temperature conditions: group 3 has good water stability, low-temperature cracking resistance, and workability. Although its strength and high-temperature performance are slightly insufficient, it is more suitable for areas with low annual average temperature; Without considering workability, group 9 has excellent high-temperature performance, although its low-temperature cracking resistance is slightly insufficient, it is more suitable for areas with high annual average temperature. The recommended results are shown in Table 15.

The research ultimately determined the key optimization parameters for the CPAM: when the total filler amount is 4.3%, the comprehensive performance is the best. An excessive or insufficient amount may affect the construction workability as well as the temperature performance. The ratio of bentonite and cement replacing the mineral powder is optimal at 50%, which can significantly improve the water stability, strength, and high-temperature performance. The ratio of bentonite to cement should be controlled at 0.2:1, as an excessive amount of bentonite will damage the low-temperature cracking resistance.

### 4.5. Analysis of Asphalt Film Morphology and Mechanism

**Figure 11:**
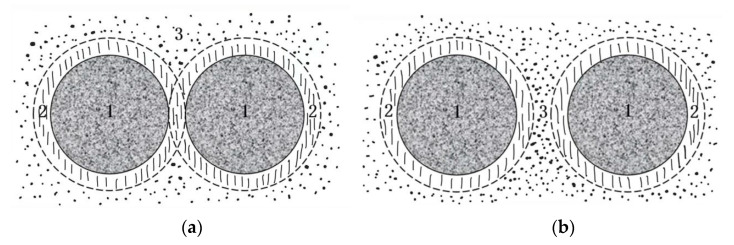
The asphalt film in CPAM. (**a**) Structure asphalt connection. (**b**) Free asphalt connection. Note: 1 represents aggregates; 2 represents structural asphalt; 3 represents free asphalt.

The filler, as the dispersed phase, not only affects the rheological and mechanical properties of the continuous phase asphalt binder but also significantly influences the road performance of asphalt mixtures, such as rutting, fatigue, water erosion, and low-temperature cracking [21,26]. The asphalt slurry formed by the filler and asphalt interacts with the mineral surface, and the adhesion of mixture comes from the cohesion of asphalt and the adhesion of asphalt slurry to the mineral. Asphalt molecules form a “structural asphalt” adsorption film on the surface of aggregates, while the asphalt that does not interact with the surface of the aggregates is “free asphalt”. As shown in Figure 11, the structural asphalt adheres to each other, enhancing the cohesion of the mixture. However, when the asphalt amount is too large, the free asphalt is over-represented. The lubricating effect will cause the aggregate particles to slide, reducing the strength of the mixture. The specific surface area of the filler can reach 80% of the total aggregate surface area, significantly affecting the ratio of structural asphalt to free asphalt [27,28,29], and the active fillers such as cement can further enhance the strength of the asphalt paste through hydration reactions. Therefore, optimizing the types and proportions of fillers can effectively improve the performance of CPAM.

**Figure 12:**
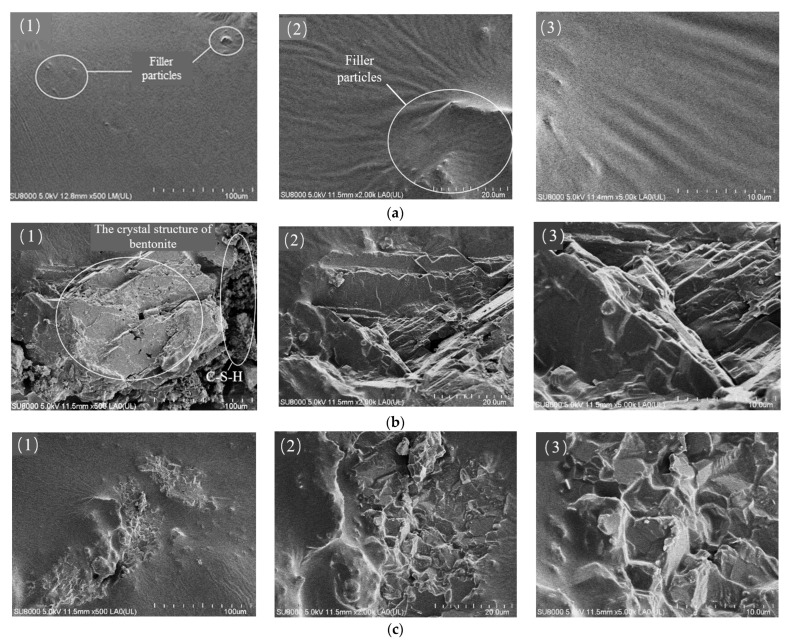
SEM images of asphalt slurry before and after freeze–thaw cycle. (**a1**) 500× scanning image before freeze–thaw cycle; (**a2**) 2000× scanning image before freeze–thaw cycle; (**a3**) 5000× scanning image before freeze–thaw cycle; (**b1**) 500× scanning image after freeze–thaw cycle (bentonite crystals); (**b2**) 2000× scanning image after freeze–thaw cycle (bentonite crystals); (**b3**) 5000× scanning image after freeze–thaw cycle (bentonite crystals); (**c1**) 500× scanning image after freeze–thaw cycle (hydrated calcium silicate); (**c2**) 2000× scanning image after freeze–thaw cycle (hydrated calcium silicate); (**c3**) 5000× scanning image after freeze–thaw cycle (hydrated calcium silicate).

The surface microstructure of the CPAM before and after freeze–thaw was observed using SEM. The dispersion and mortar condition of cement and bentonite in the asphalt were analyzed. The results are shown in Figure 12, where (1), (2), and (3) were magnified 500×, 2000×, and 5000×, respectively.

As can be seen from Figure 12(a1), the surface of the mixture before the freeze–thaw cycle was smooth and flat, and the filler particles formed a uniform distribution of asphalt mortar structure. When magnified 2000 times, it can be observed that the filler particles buried inside the asphalt cause wrinkles at the edges of the protruding small particles from Figure 12(a2).

Observing Figure 12(b1), the mineral crystal structure of the main body was similar to the lamellar interlayer structure of bentonite, which was presumed to be the product of the expansion of bentonite after absorbing water, and the flocculant on the right side was presumed to be the product of the hydration of cement. From the volume size, it can be judged that the volume of bentonite after water absorption is much larger than that of the cement hydration product. From Figure 12(b2,b3), it can be seen that the asphalt film on the surface of the CPAM after freeze–thaw had been damaged, the asphalt film had become rough, and some open pores appeared. The reason for this may be that water seeped into the damaged asphalt film and came into contact with cement and bentonite, the volume of bentonite expanded due to water absorption, and cement reacted with water to form hydration products. With localized magnification to 2000 times and 5000 times, the concave and convex structure can be seen more clearly, which is similar to the C-S-H gel structure observed by Belkowitz [30]. The above conclusion proves that after adding bentonite and cement to the CAPM, the hydration products of the fillers can form a stable crosslinked gel structure between the aggregates, strengthen the adhesion between the mixtures, and reduce the voids, which in turn improves the road performance of the CAPM.

## 5. Conclusions

Two kinds of fillers, bentonite and cement, were selected to replace part of the mineral powder to optimize the ratio of CPAM, and the mechanism of the filler’s influence on the performance of the mixture was analyze, resulting in the following conclusions:

(1) According to the results of the range analysis and ANOVA, the total filler amount significantly affected the temperature performance and workability of CPAM and influenced the CPAM’s early strength and water stability to some extent; the ratio of replacing mineral powder significantly impacted the water stability, but it had no obvious effect on the high-temperature performance and strength; and the ratio of bentonite and cement significantly influenced the low-temperature cracking resistance, without affecting other performance.

(2) Through single factor ANOVA, the optimal filler ratio was finally determined as follows: the total filler amount was 4.3%, the ratio of bentonite and cement replacing the mineral powder was 50%, and the ratio of bentonite to cement was 0.2:1. The forming strength, residual stability after immersion, and TSR of CPAM using the optimal filler ratio increased by 7.37%, 20.95%, and 17.15%, respectively.

(3) The fillers could affect the ratio of structural asphalt and free asphalt in the mixture. The structure of bentonite after absorbing water and the products of cement hydration can form a stable cross-linked gel system between the aggregates, strengthening the adhesion between the mixtures and reducing the voids in mixture, thereby improving the road performance of CPAM.

## Figures and Tables

**Table 1:** SK-90# petroleum asphalt performance technical indicators.

Technical Indicator	Unit	Measured Value	Specification Requirement	Test Method
Penetration	0.1 mm	87	80–100	T0604
Pointness Index	-	1.0	−1.8–1.0	T0604
Ductility (10 °C)	cm	27	≥20	T0605
Ductility (15 °C)		100	≥100	T0605
Softening point (R&B)	°C	45	≥43	T0606
Wax content (distillation)	%	1.4	≤3.0	T0615
Power viscosity (60 °C)	Pa·s	170	≥160	T0625
Density (15 °C)	g/cm^3^	0.95	Measured value	T0603
After RTFOT				
Weight change	%	−0.1	≤±0.8	T0609
Residual penetration ratio (25 °C)		73	≥54	T0604
Residual ductility (10 °C)	cm	9	≥6	T0605
Softening point incremental	°C	3.2	-	T0606
Viscosity ratio (60 °C)	-	1.54	-	T0625

**Table 2:** Limestone aggregate technical indicators.

Technical Indicator	Unit	Measured Value		Specification Requirement	Test Method
		Limestone coarse aggregates			
		4.75–9.5 mm	9.5–13.2 mm		
Crushing value	%		13.2	≤26.0	T0316
Abrasion value			12.7	≤28.0	T0317
Mud content			0.78	≤3.00	T0314
Adhesion to asphalt			Grade 5	Greater than grade 4	T0616
Apparent density	g∙cm^−3^	2.827	2.837	≥2.600	T0304
Bulk density		2.746	2.766		T0304
Needle-flake content	%	9.4	6.1	≤18.0; ≤12.0	T0312
Water absorption		0.77	0.51	≤2.00	T0304
		Limestone fine aggregates			
		0–2.36 mm	2.36–4.75 mm		
Apparent density	g∙cm^−3^	2.743	2.853	≥2.500	T0304
Bulk density		2.665	2.767		T0304
Needle-flake content	%		1.9	≤3.0	T0304

**Table 3:** Limestone mineral powder technical indicators.

Technical Indicator		Unit	Measured Value	Specification Requirement	Test Method
Apparent relative density		g∙cm^−3^	2.807	≥2.500	T0352
Particle size range	0.6	mm	100	100	T0351
	0.15		98.70	90–100	
	0.075		90.30	75–100	
Hydrophilic coefficient		%	0.75	<1	T0353
Moisture content			0.2	≤1	T0305
Specific surface area		m^2^∙kg^−1^	438	≥400	GB/T 8074 [18]

Note: Mineral powder specific surface area testing referenced GB/T 18046-2017 [19].

**Table 4:** Cement main technical indicators.

Technical Indicator	Unit	P.O 42.5	National Standard
MgO	%	1.8	≤5.0
SO_3_		2.11	≤3.5
Heat loss		3.89	≤5
Chloridion		0.018	≤0.06
Specific surface area	m^2^∙kg^−1^	364	≥300

**Table 5:** Bentonite main technical indicators.

Technical Indicator	Unit	Measured Value
Specific gravity	%	2.74
Liquid limit		180
Plastic limit		51
Plasticity index		128
Swelling index	mL∙2 g^−1^	18.2

**Table 6:** Orthogonal test combination table.

	Factors	A *	B *	C *
Level				
1		2.3	10	0.2:1
2		4.3	30	0.6:1
3		6.3	50	1:1

* A represents total filler amount, %; B represents proportion of bentonite and cement replacing mineral powder, %; C represents the ratio of bentonite and cement.

**Table 7:** The percentage of each of the three materials in the orthogonal group.

Number	Total Filler Usage	Mineral Powder	Bentonite	Cement
Unit	%			
1	2.3	90.0	1.7	8.3
2	2.3	70.0	15.0	15.0
3	2.3	50.0	18.8	31.2
4	4.3	90.0	5.0	5.0
5	4.3	70.0	11.3	18.7
6	4.3	50.0	8.3	41.7
7	6.3	90.0	3.7	6.3
8	6.3	70.0	5.0	25.0
9	6.3	50.0	25.0	25.0

**Table 8:** CPAM orthogonal test results summary.

No.	Strength		Workability	Water Stability		Low-Temperature Performance		High-Temperature Performance
	Early Strength	Forming Strength	Scattering Time	Residual Stability	TSR	Failure Bending and Tensile Strain	Failure Bending and Tensile Strength	Dynamic Stability
Unit	kN	kN	s	%		*ε*_B_/μ*ε*	*R*_B_/MPa	Time/mm
1	2.07	7.22	40	88.0	88.4	4235	4.03	2698
2	1.92	7.09	42	86.5	84.6	4008	3.98	2677
3	1.77	7.56	47	94.2	90.6	3523	4.55	3120
4	2.01	7.78	75	85.3	92.0	3625	5.15	4018
5	2.1	7.98	89	103.2	88.7	3314	5.34	4320
6	2.49	8.89	83	105.8	93.5	2870	6.21	4179
7	2.58	7.47	204	75.7	92.2	2914	5.76	3516
8	2.22	8.61	195	82.3	90.2	2533	5.23	3432
9	2.26	7.73	289	90.4	90.3	1984	4.32	4213

**Table 9:** Range analysis table for cold patching materials.

Indicator	Unit	Factor	*K*_1_	*K*_2_	*K*_3_	*k*_1_	*k*_2_	*k*_3_	*R*
Early strength	kN	A	5.76	6.60	7.06	1.92	2.20	2.35	0.43
		B	6.66	6.24	6.52	2.22	2.08	2.17	0.14
		C	6.78	6.19	6.45	2.26	2.06	2.15	0.20
Forming strength	kN	A	21.87	24.65	23.81	7.29	8.22	7.94	0.93
		B	22.40	23.68	24.18	7.49	7.89	8.06	0.57
		C	24.72	22.60	23.01	8.24	7.53	7.67	0.71
Scattering time	s	A	129.00	247.00	688.00	43.00	82.33	229.33	186.33
		B	319.00	326.00	419.00	106.33	108.67	139.67	33.33
		C	318.00	406.00	340.00	106.00	135.33	113.33	29.33
Residual stability	%	A	268.72	294.30	248.40	89.57	98.10	82.80	15.30
		B	248.97	272.03	290.42	82.99	90.68	96.81	13.82
		C	276.10	262.21	273.12	92.03	87.40	91.04	87.25
TSR	%	A	263.56	269.37	272.71	87.85	89.79	90.90	3.05
		B	267.81	263.47	274.35	89.27	87.82	91.45	3.63
		C	272.01	262.13	271.50	90.67	87.38	90.50	3.29
Failure bending and tensile strain	$\varepsilon_{B}$∙με^−1^	A	11,766.0	9809.00	7431.00	3922.00	3269.70	2477.00	1445.00
		B	10,774.0	9855.00	8377.00	3591.33	3285.00	2792.30	799.00
		C	9638.00	9617.00	9751.00	3212.67	3205.67	3250.33	44.67
Dynamic stability	time∙mm^−1^	A	8495.0	12,517.0	11,161.0	2831.67	4172.33	3720.33	1340.67
		B	10,232.0	10,429.0	11,512.0	3410.67	3476.33	3837.33	426.67
		C	10,309.0	10,908.0	10,956.0	3436.33	3636.00	3652.00	215.67

**Table 10:** Analysis of the extreme differences influence order in cold-patch performance tests.

Performance	Unit	Indicator	Order of Influence
Strength	kN	Early strength	A > C > B
		Forming strength	A > C > B
Workability	s	Scattering time	A > B > C
Water stability	%	Residual stability	A > B > C
		TSR	B > C > A
Low-temperature performance	$\varepsilon_{B}$∙με^−1^	Failure bending and tensile strain	A > B > C
High-temperature performance	time∙mm^−1^	Dynamic stability	A > B > C

**Table 11:** Results of ANOVA for orthogonal tests of CPAM.

Indicator	Unit	Source of Variance	*S_j_*	*d_f_*	*MS*	*MS* After Combining Deviations	*F*	*P*
Early strength	kN	A	0.29	2	0.145	0.145	3.434	0.1014
		B	0.03	2	0.015	—	—	—
		C	0.06	2	0.029	—	—	—
		D deviation	0.16	2	0.082	0.042	—	—
Forming strength	kN	A	1.36	2	0.678	0.678	7.815	0.1134
		B	0.52	2	0.258	0.258	2.972	0.2518
		C	0.84	2	0.421	0.421	4.861	0.1706
		D deviation	0.17	2	0.087	0.087	—	—
Scattering time	s	A	57,876.22	2	28,938.110	28,938.11	33.769	0.0031
		B	2077.56	2	1038.780	1038.780	1.212	0.3877
		C	1398.22	2	699.110	—	—	—
		D deviation	2029.56	2	1014.780	856.940	—	—
Residual stability	%	A	352.67	2	176.336	176.336	6.788	0.0518
		B	287.55	2	143.777	143.777	5.535	0.0705
		C	35.61	2	17.805	—	—	—
		D deviation	68.30	2	34.149	25.977	1.000	—
TSR	%	A	14.31	2	7.156	7.156	8.975	0.1002
		B	20.00	2	10.000	10.000	12.543	0.0738
		C	20.64	2	10.318	10.318	12.943	0.0717
		D deviation	1.59	2	0.797	0.797	1.000	
Failure bending and tensile strain	$\varepsilon_{B}$∙με^−1^	A	3,141,884.20	2	1,570,942.110	1,570,942.11	426.912	0.0023
		B	974,961.55	2	487,480.780	487,480.780	132.476	0.0075
		C	3462.89	2	1731.440	—	—	—
		D deviation	11,256.22	2	5628.110	3679.778	—	—
Dynamic stability	time∙mm^−1^	A	2,791,419.60	2	1,395,709.800	1,280,643.100	23.124	0.0063
		B	316,677.56	2	158,338.800	110,272.100	1.991	0.2511
		C	86,634.89	2	43,317.400	—	—	—
		D deviation	134,889.56	2	67,444.8	55,381.1	1.000	0.5000

**Table 12:** Significance analysis of ANOVA.

Factor	Early Strength	Forming Strength	Scattering Time	Residual Stability	TSR	Failure Bending and Tensile Strain	Dynamic Stability
Unit	kN		s	%		$\varepsilon_{B}$∙μƐ^−1^	Time∙mm^−1^
A	RS *	HI *	ES *	RS	RS	ES	ES
B	None	None	None	RS	RS	ES	None
C	None	HI	None	None	RS	None	None

* RS represents relatively significant; ES represents extremely significant; HI represents has influence.

**Table 13:** Performance comparison of cold-patching materials.

Performance	Early Strength	Forming Strength	Scattering Time	Residual Stability	TSR	Failure Bending and Tensile Strain	Dynamic Stability
Unit	kN		s	%		$\varepsilon_{B}$∙μƐ^−1^	Time∙mm^−1^
Group 4	2.01	7.78	75	85.3	92.0	3625	4018
Group 5	2.1	7.98	89	103.2	88.7	3314	4320
Group 6	2.49	8.89	83	105.8	93.5	2870	4179

**Table 14:** Significance analysis table.

Performance	Early Strength	Forming Strength	Scattering Time	Residual Stability	TSR	Failure Bending and Tensile Strain	Dynamic Stability
Unit	kN		s	%		$\varepsilon_{B}$∙μƐ^−1^	Time∙mm^−1^
Group 6	2.49	8.89	83	105.8	93.5	2870	4179
Conventional group	2.48	8.28	78	87.5	79.8	3144	4261

**Table 15:** Optimal combination of CPAM.

Group	Total Filler Dosage	Ratio of Bentonite and Cement to Replace Mineral Powder	Bentonite: Cement	Summary
6	4.3%	50%	0.2:1	Optimal comprehensive performance
3	2.3%	50%	0.6:1	Poor high-temperature resistance, suitable for low-temperature regions
9	6.3%	50%	1:1	Poor low-temperature resistance, suitable for high-temperature regions

## Data Availability

The data presented in this study are available on request from the corresponding author. The data are not publicly available due to privacy restrictions.

## Abbreviations

The following abbreviations are used in this manuscript:

CPA	Cold-patched asphalt
CPAM	Cold-patch asphalt mixture
SEM	Scanning electron microscope
